# *Mgat1*-dependent N-glycosylation of Membrane Components Primes *Drosophila melanogaster* Blood Cells for the Cellular Encapsulation Response

**DOI:** 10.1371/journal.ppat.1002819

**Published:** 2012-07-19

**Authors:** Nathan T. Mortimer, Balint Z. Kacsoh, Erin S. Keebaugh, Todd A. Schlenke

**Affiliations:** Department of Biology, Emory University, Atlanta, Georgia, United States of America; Stanford University, United States of America

## Abstract

In nature, larvae of the fruitfly *Drosophila melanogaster* are commonly infected by parasitoid wasps, and so have evolved a robust immune response to counter wasp infection. In this response, fly immune cells form a multilayered capsule surrounding the wasp egg, leading to death of the parasite. Many of the molecular mechanisms underlying this encapsulation response are conserved with human immune responses. Our findings suggest that protein N-glycosylation, a common protein post-translational modification of human immune proteins, may be one such conserved mechanism. We found that membrane proteins on Drosophila immune cells are N-glycosylated in a temporally specific manner following wasp infection. Furthermore we have identified mutations in eight genes encoding enzymes of the N-glycosylation pathway that decrease fly resistance to wasp infection. More specifically, loss of protein N-glycosylation in immune cells following wasp infection led to the formation of defective capsules, which disintegrated over time and were thereby unsuccessful at preventing wasp development. Interestingly, we also found that one species of Drosophila parasitoid wasp, *Leptopilina victoriae*, targets protein N-glycosylation as part of its virulence mechanism, and that overexpression of an N-glycosylation enzyme could confer resistance against this wasp species to otherwise susceptible flies. Taken together, these findings demonstrate that protein N-glycosylation is a key player in Drosophila cellular encapsulation and suggest that this response may provide a novel model to study conserved roles of protein glycosylation in immunity.

## Author Summary

Organisms such as the fruitfly Drosophila melanogaster have long been used as model systems to understand complex aspects of human biology. Work on Drosophila antimicrobial immunity has led to identification of mechanisms underlying human innate immunity, such as the use of Toll-like receptors for recognizing antigen and initiating humoral immune responses. Flies and humans are also infected by larger parasites against which they mount immune blood-cell based responses, but the genetic basis for cellular immunity is poorly characterized. In nature, flies are often infected by parasitoid wasps that lay their eggs in fly larvae, inducing a cellular immune response in the flies. Fly blood cells surround the wasp egg and form a tightly connected capsule leading to death of the egg in a process called encapsulation, which is similar to human granuloma formation. In this study we identified eight new genes that are important for encapsulation. These genes are part of the N-glycosylation pathway, and we found that without N-glycosylation of proteins on blood cell surfaces, capsules surrounding wasp eggs cannot consolidate into a tight capsule, allowing the wasps to escape. Interestingly, we also found a wasp that disrupts N-glycosylation so that it can evade the encapsulation response. Our work may provide a model to better understand the role of N-glycosylation in human immunity.

## Introduction

Cellular encapsulation of invading organisms is a vital and conserved aspect of insect immunity [1], [2]. In this immune response the invader is recognized as foreign and then surrounded by a multilayered capsule of immune cells which serves to kill or sequester it. Capsule formation can be targeted against any foreign object too large to be phagocytosed, including parasitoid wasp eggs and wasp larvae, fungal and protozoan parasites, and even abiotic objects [1]. Cellular encapsulation represents an important aspect of resistance against pathogens in insect vectors of human disease [3], [4] and in the genetic model organism *Drosophila melanogaster* [5], [6] and is functionally similar to granuloma formation in vertebrates [7].

In nature, Drosophila are commonly parasitized by a wide range of parasitoid wasps [8]. Among these are larval parasitoids, which attack fly larvae and inject their eggs and venom directly into the larval hemocoel. While flies mount a robust cellular encapsulation response against parasitoid eggs, the wasps' venom contains factors that target host immune mechanisms and allow wasps to evade or suppress capsule formation of their natural hosts [9], [10]. In Drosophila, cellular encapsulation is accompanied by capsule melanization, and is mediated by immune cells known as hemocytes. Flies have three subtypes of hemocytes: macrophage-like plasmatocytes, which act as immune sentinels and comprise ∼95% of all hemocytes in unattacked larvae; crystal cells, which contain the melanization machinery and comprise ∼5% of all hemocytes in unattacked larvae; and lamellocytes, large flattened cells which function in encapsulation and are induced following parasitoid attack and during pupation [11], [12].

Egg encapsulation begins when plasmatocytes recognize the wasp egg as foreign and adhere to it. Newly differentiated lamellocytes then bind to this plasmatocyte layer. Subsequent lamellocytes adhere to each other to form a consolidated multilayered capsule around the melanized inner layers, leading to death of the wasp egg [1], [13]. Genetic and transcriptional analyses have begun to provide insight into the regulation of the encapsulation response [14], [15], [16], [17], [18]. Despite this progress, the molecular mechanisms underlying the process of encapsulation are still largely unexplored.

Cellular encapsulation has long been studied in Drosophila melanotic tumor (*tu*) mutants, a class of mutants that are characterized by precocious lamellocyte differentiation and the encapsulation of self tissues [19], [20], [21]. Studies of various *tu* mutants have revealed the specific binding of the lectin wheat germ agglutinin (WGA) to lamellocyte surfaces during conditions promoting self-encapsulation [22], [23]. WGA recognizes N-acetylglucosamine (GlcNAc) residues in protein-linked glycans [24]. The WGA binding observed in *tu* mutants suggests that protein glycosylation of lamellocyte membrane proteins is increased during the encapsulation response, although a functional role of protein glycosylation in encapsulation has never been demonstrated.

Protein glycosylation is one of the most abundant post-translational modifications and plays a variety of roles in protein regulation and recognition [25]. There are two main classes of protein glycosylation, Asn- or N-linked glycans, and Ser-/Thr- or O-linked glycans. WGA has been shown to recognize N-glycosylated proteins found in the plasma membrane or secreted into the extracellular space [26], [27], [28], and O-GlcNAc glycans which are exclusively found intracellularly [27], [29]. The cell surface WGA staining observed in *tu* mutants therefore likely reflects the presence of N-glycosylated proteins.

The N-linked glycosylation pathway [reviewed in 28] begins with the biosynthesis of a dolichol-linked precursor molecule which is then transferred to an Asn residue in the target protein by oligosaccharyltransferase (Ost). The precursor molecule is then trimmed by the activity of the alpha-glucosidase enzymes to produce oligomannose-type N-glycans. These structures can be further modified by alpha-mannosidase-I and β1,2-*N*-acetylglucosaminyltransferase I (GnTI). GnTI activity initiates the synthesis of hybrid, paucimannose and complex-type N-glycans by adding an N-acetylglucosamine residue to the glycan core. This produces a hybrid-type N-glycan, which can be further modified by a variety of enzymes.

Protein N-glycosylation has been biochemically characterized in Drosophila at multiple life stages and in a variety of glycosylation defective mutant backgrounds [30], [31], [32], [33], [34], [35], [36], [37]. Additional genetic characterization has revealed roles for N-glycosylation in tissue morphogenesis, nervous system development, adult locomotion, oxidative stress resistance, and lifespan regulation [31], [38], [39], [40]. Based on the appearance of WGA positive glycosylated proteins on encapsulating lamellocytes, we hypothesized that the N-linked protein glycosylation pathway was also required for wasp resistance in Drosophila.

## Results/Discussion

### WGA staining of Drosophila lamellocytes is temporally dynamic following wasp parasitization

**Figure 1:**
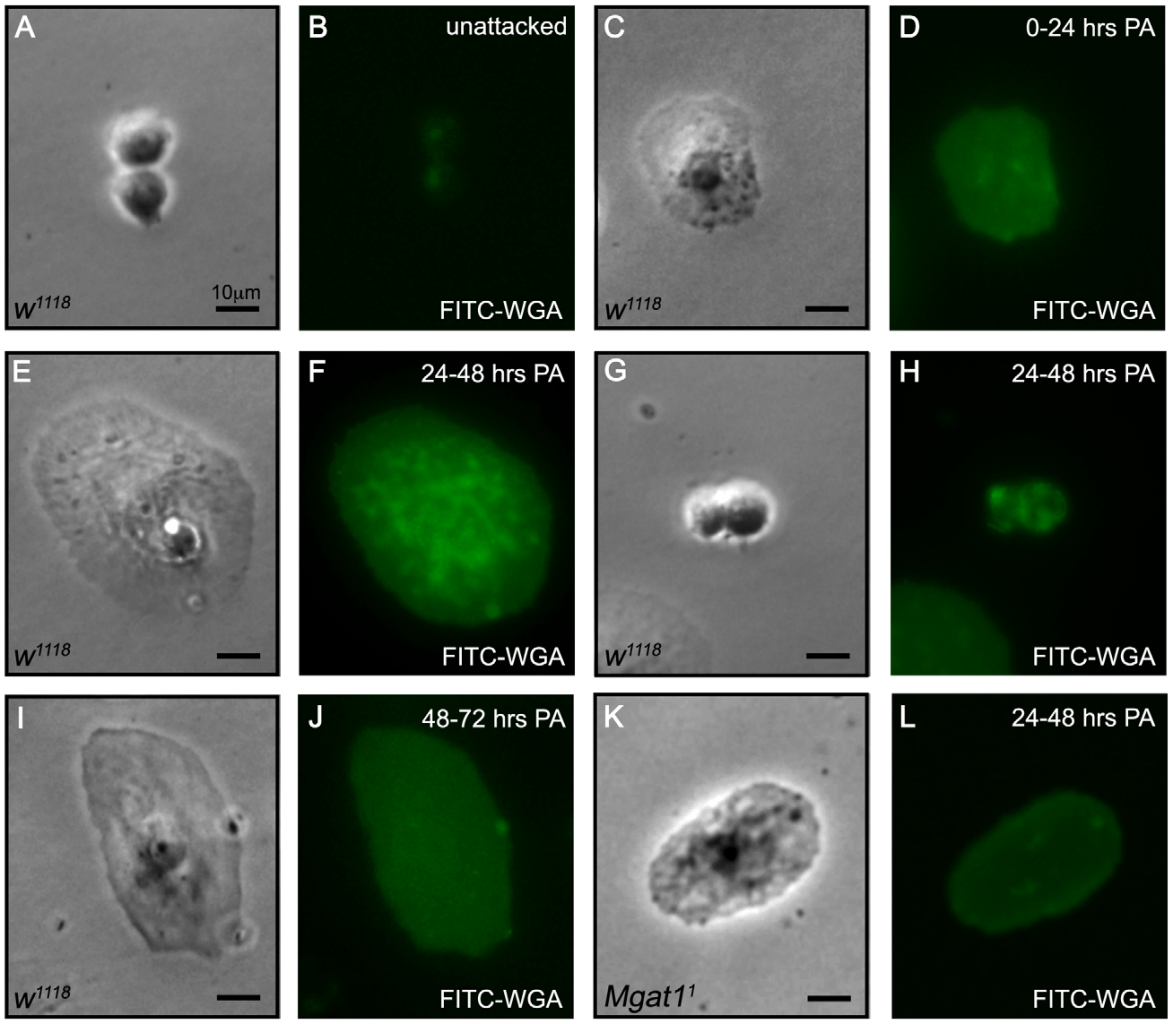
N-glycosylation of hemocytes is temporally specific following wasp attack. Paired brightfield and FITC images of hemocytes stained with FITC-WGA at the indicated time after wasp attack reveals that WGA staining of hemocytes is dynamic following wasp attack. (A,B) Plasmatocytes from unattacked *w^1118^* larvae, age matched with the 24–48 hour post attack (PA) time point, did not stain with WGA. (C,D) At 0–24 hours PA, lamellocytes from *L. clavipes* attacked *w^1118^* larvae began to show WGA staining. (E–H) A strong, speckled, WGA staining pattern was seen in lamellocytes (E,F), and to a lesser extent, plasmatocytes (G,H) from attacked *w^1118^* larvae at 24–48 hours PA. (I,J) By 48–72 hours PA, lamellocytes from attacked *w^1118^* larvae no longer stained with WGA. (K,L) Lamellocytes from attacked *Mgat1^1^* mutant larvae did not stain with WGA at 24–48 hours PA, demonstrating that the WGA signal is dependent on N-glycosylation pathway function. Scale bar in all panels indicate 10 µm.

To assay glycosylation state, we stained hemocytes from attacked and unattacked larvae with fluorescein isothiocyanate (FITC) labeled WGA. In accordance with previous findings [22], we found that hemocytes from unattacked larvae were negative for WGA staining (WGA-; Figure 1A,B). We then assayed WGA staining following attack by *Leptopilina clavipes*, a parasitoid wasp from the family Figitidae that provokes an immune response in attacked larvae and is avirulent with respect to *D. melanogaster* [8]. Hemocytes were stained and observed at the following three time points: 0–24 hours, during which time plasmatocytes surrounded the wasp egg and lamellocyte differentiation began; 24–48 hours when lamellocyte-mediated encapsulation and capsule melanization occurred; and at 48–72 hours when melanotic encapsulation was complete.

We found that lamellocytes dissected 0–24 hours post-attack (PA) showed low-level WGA staining (Figure 1C,D), while plasmatocytes were still WGA−. In contrast, lamellocytes dissected at 24–48 hours PA showed a strong, speckled WGA staining pattern (WGA+; Figure 1E,F), reminiscent of that seen in *tu* mutant larvae [22], [23]. Additionally, WGA+ plasmatocytes were occasionally observed at this time point (Figure 1G,H). Hemocytes dissected from larvae following the completion of melanotic encapsulation (48–72 hours PA) were WGA− (Figure 1I,J). Therefore, the WGA+ phenotype was specific for lamellocytes during the period of encapsulation. These results paralleled previous studies of *tu(1)Sz*, a conditional self-encapsulating *tu* mutant strain. In *tu(1)Sz*, WGA+ lamellocytes were found in larvae that were actively encapsulating either self or transplanted foreign tissues, but not in larvae raised in non-encapsulating conditions [22]. In combination, these findings suggest that WGA+ lamellocytes are exclusively found in larvae undergoing active encapsulation.

### WGA staining of lamellocytes reflects protein N-glycosylation

To determine whether this WGA+ phenotype reflects the presence of N-glycosylated proteins, we analyzed the WGA staining pattern of lamellocytes from mutant larvae with demonstrated defects in N-glycosylation. A null allele of *Mgat1*, the locus that encodes the homolog of the GnTI enzyme [41], has been demonstrated to induce altered patterns of N-glycosylation [31]. We stained hemocytes from *L. clavipes* attacked *Mgat1^1^* mutant larvae at 24–48 hours PA. Hemocyte subtypes were easily distinguishable in *Mgat1^1^* mutant larvae on the basis of morphology and size and did not differ from wild type cells (as described in [11], data not shown). We found that mutant lamellocytes had greatly reduced WGA staining (Figure 1K,L). This demonstrated that the dynamic protein glycosylation revealed by WGA staining of lamellocytes is dependent on function of the N-glycosylation pathway, and therefore suggests that WGA is recognizing N-glycosylated proteins on lamellocytes.

### N-glycosylation mutants show impaired encapsulation ability

**Figure 2:**
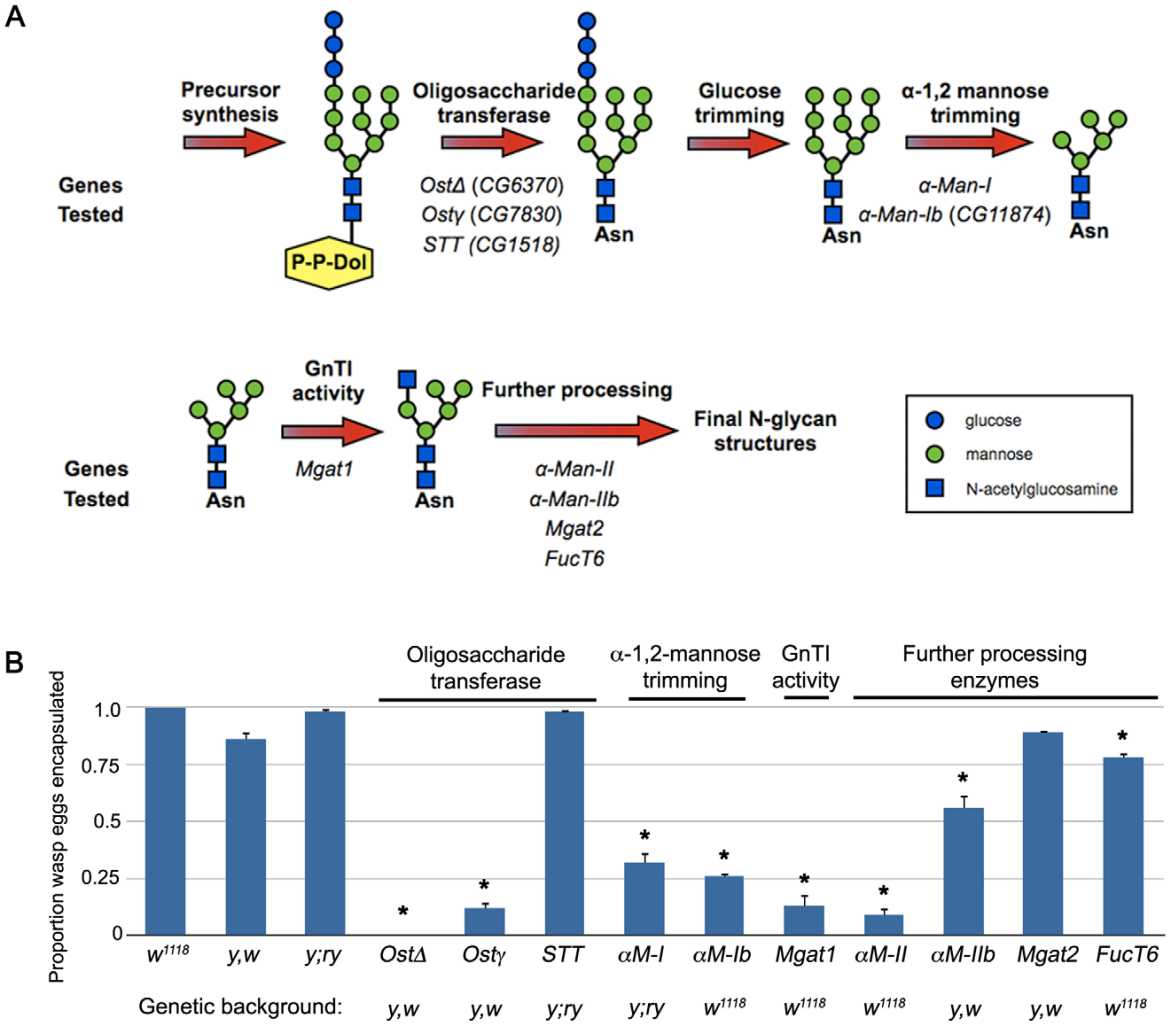
Multiple N-glycosylation pathway mutants fail to encapsulate *L. clavipes* eggs. (A) Schematic representation of the N-glycosylation pathway [adapted from 65]. The Drosophila orthologs of pathway members tested by mutant analysis are indicated and grouped according to function. Structures are drawn according to [28]. (B) Proportion of *L. clavipes* eggs encapsulated in the indicated genotypes, demonstrating encapsulation deficiency of multiple pathway mutants. Error bars indicate standard error of the mean; * indicates p<0.01 compared to indicated genetic background control; α-M-I: α-Man-I; α-M-Ib: α-Man-Ib; α-M-II: α-Man-II; α-M-IIb: α-Man-IIb.

To test the hypothesis that protein N-glycosylation is required for the cellular encapsulation response, we assayed wasp egg encapsulation rates in larvae with mutations in predicted N-glycosylation pathway genes [pathway illustrated in Figure 2A, Drosophila homolog predictions from the KEGG database, 42]. We exposed larvae to the avirulent wasp *L. clavipes*, which induces a strong cellular encapsulation response in *D. melanogaster* following attack, and scored egg encapsulation rate (results shown in Figure 2B). We found that the wild type background control strains (*w^1118^*, *y*,*w* and *y*;*ry*) successfully encapsulated *L. clavipes* eggs (*w^1118^*: 100% encapsulated, n = 90 eggs; *y*,*w*: 85.5% encapsulated, n = 117 eggs; *y*;*ry*: 97.6% encapsulated, n = 125 eggs). We next tested mutations targeting the oligosaccharide transferase (Ost), α-1,2-mannose trimming and GnTI steps in the N-glycosylation pathway, along with a variety of mutations in enzymes in downstream N-glycan processing pathways. Mutations in the Δ and γ subunits of Ost (*OstΔ*, encoded by *CG6370* and *Ostγ*, encoded by *CG7830*), which are expected to abolish protein N-glycosylation, led to an almost complete inability to encapsulate wasp eggs (*OstΔ*: 0% encapsulated, n = 83 eggs, p<0.001 relative to *y*,*w* control; *Ostγ*: 12.0% encapsulated, n = 92 eggs, p<0.001 relative to *y*,*w* control). Mutation of *CG1518*, one homolog of the STT3 subunit of Ost, had no effect on encapsulation (98.0% encapsulated, n = 98 eggs, p = 0.89 relative to *y*;*ry* control). This suggests either that the STT3 subunit is not required for encapsulation or that another locus is redundant to *CG1518* in this process.

Mutations in the α-1,2-mannosidase homologs *α-Man-I* and *CG11874* (referred to here as *α-Man-Ib*) displayed an intermediate encapsulation phenotype (*α-Man-I*: 32.4% encapsulated, n = 102 eggs, p<0.001 relative to *y*;*ry* control; *α-Man-Ib*: 26.3% encapsulated, n = 114 eggs, p<0.001 relative to *w^1118^* control). This suggests a degree of redundancy between these genes and possibly a third α-1,2-mannosidase homolog (*CG31202*), perhaps explaining previous observations [30]. *Mgat1^1^* mutant larvae were significantly impaired in their encapsulation ability (13.4% encapsulated, n = 97 eggs, p<0.001 relative to *w^1118^* control), suggesting that hybrid, or more highly processed types of N-glycan structures are required. We then assayed encapsulation rates in downstream *Mgat1*-dependent processing steps, and found that *α-Man-II* mutants were also impaired in encapsulation ability (8.9% encapsulated, n = 79 eggs, p<0.001 relative to *w^1118^* control). Mutants in the additional downstream N-glycan processing enzymes *α-Man-IIb* (55.5% encapsulated, n = 137 eggs, p = 0.009 relative to *y*,*w* control) and *FucT6* (77.9% encapsulated, n = 86 eggs, p<0.001 relative to *w^1118^* control) displayed only minor decreases in encapsulation rate, suggesting either genetic redundancy with additional, untested α-mannosidase and fucosyl transferase loci for these enzymes, or that their products play minor roles in encapsulation.

A mutation in *Mgat2* had no effect on wasp egg encapsulation (89.0% encapsulated, n = 91 eggs, p = 0.41 relative to *y*,*w* control). The protein-acetylglucosaminyltransferases *Mgat1* and *Mgat2* encode unique orthologs of GnTI and GnTII (respectively) and act in a sequential manner to process N-linked glycans. The differential requirement for encapsulation suggests that *Mgat1*-dependent hybrid N-glycans but not *Mgat2*-dependent complex N-glycans are important for cellular encapsulation.

The impaired immune response due to loss of function mutations in multiple N-glycosylation genes suggests that the protein N-glycosylation revealed by WGA+ lamellocytes is essential for cellular encapsulation of parasitoid wasp eggs. Interestingly, increased WGA staining of hemocytes has been observed in other insects following parasitization [43], [44], suggesting that N-glycosylation plays a conserved role in encapsulation-mediated immune responses in insects.

### *Mgat1* is haploinsufficient for cellular encapsulation

Of the alleles tested in this survey, only *Mgat1^1^* has been biochemically characterized, and these mutants were found to be deficient in N-glycosylation activity [31]. Therefore, we decided to focus further experiments on *Mgat1* in order to fully characterize the role of protein N-glycosylation in the cellular encapsulation process.

Surprisingly we found that larvae heterozygous for *Mgat1^1^* (in trans to a *CyO-GFP* balancer) were also deficient in encapsulation (3.2% encapsulated, n = 31 eggs). The phenotype seen in these heterozygotes suggests that wasp egg encapsulation may be very sensitive to protein N-glycosylation levels; GnTI enzyme activity is reduced to approximately 40% of wild type activity in *Mgat1^1^/+* flies, but they are wild type with respect to all other known *Mgat1* mutant phenotypes, including male fertility, locomotor activity, and life span [31].

**Figure 3:**
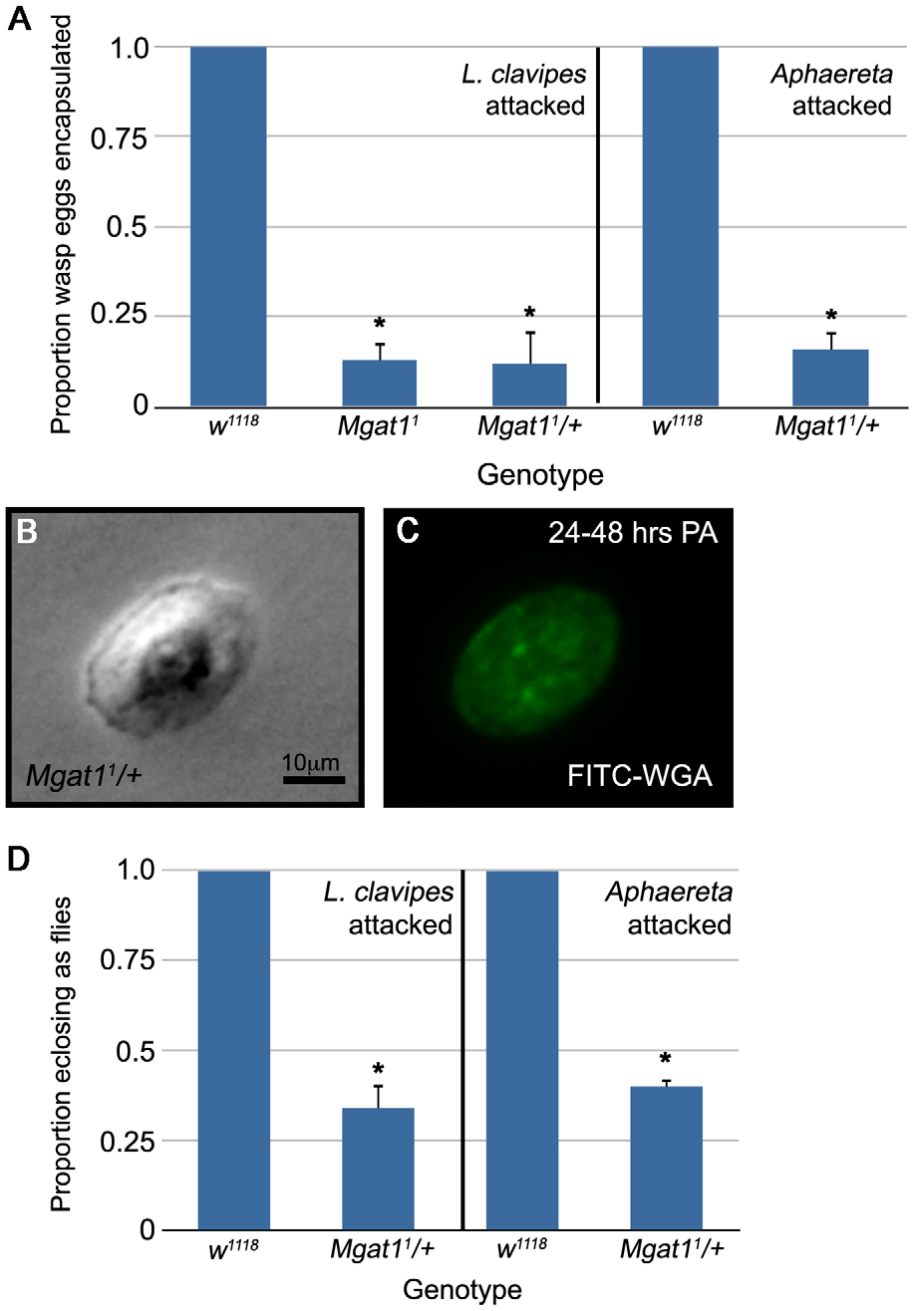
*Mgat1* is required for wasp egg encapsulation. (A) Proportion of wasp eggs encapsulated in the indicated genotypes. *Mgat1* appears haploinsufficient for encapsulation of both *L. clavipes* (left) and *Aphaereta sp.* (right) eggs. (B,C) Paired brightfield and FITC images of hemocytes stained with FITC-WGA. Lamellocytes from *L. clavipes* attacked *Mgat1^1^/+* larvae show intermediate levels of WGA staining at 24–48 hours PA. Scale bar indicates 10 µm. (D) Proportion of wasp exposed larvae of the indicated genotypes that eclosed as adult flies. The decreased encapsulation rate of *Mgat1^1^/+* larvae correlated with a decrease in fly eclosion. Error bars indicate standard error of the mean; * indicates p<0.01 relative to control.

This *Mgat1^1^* heterozygote phenotype could reflect either haploinsufficiency for the *Mgat1* locus or a synthetic interaction with the balancer chromosome. To distinguish between these possibilities, we outcrossed *Mgat1^1^* mutant males to females of the background control strain *w^1118^*. We found that these *Mgat1^1^/+* larvae also showed a profound defect in cellular encapsulation, only 12.2% of *L. clavipes* eggs were encapsulated at 48–72 hours PA (n = 49 eggs, p<0.001 relative to control; Figure 3A). The decreased encapsulation rate was independent of the gender of the mutant parent; in the reciprocal cross only 15.2% of parasite eggs were encapsulated (n = 99 eggs, p<0.001 relative to control). The *Mgat1^1^/+* phenotype is statistically indistinguishable from the *Mgat1^1^* homozygous phenotype (p = 0.90), suggesting that *Mgat1* is a haploinsufficient locus for wasp encapsulation. To avoid the larval lethality and other defects associated with *Mgat1* deficiency [31], we decided to further characterize the role of *Mgat1* in cellular encapsulation using *Mgat1^1^/+* larvae.

To test whether the decreased GnTI function in *Mgat1^1^* heterozygous larvae was sufficient to disrupt the appearance of WGA+ lamellocytes, we stained lamellocytes from *Mgat1^1^/+* larvae with FITC-WGA. *Mgat1^1^/+* lamellocytes showed a decrease in WGA staining (Figure 3B,C). The staining phenotype was intermediate between WGA+ *w^1118^* larvae and WGA− *Mgat1^1^* null larvae (compare with Figure 1E–F and K–L). This finding is in accordance with the GnTI activity seen in the respective genotypes [31]. The inability of *Mgat1^1^/+* lamellocytes with intermediate WGA staining levels to encapsulate wasp eggs suggests that lamellocyte function is extremely sensitive to glycosylation state.

### The requirement for *Mgat1*-dependent glycosylation is not specific to encapsulation of figitid wasps

The above data demonstrates a role for *Mgat1* in the encapsulation of the figitid wasp *L. clavipes*. To test whether the requirement for *Mgat1* in wasp encapsulation is specific for figitid wasps, or whether it is part of a more general encapsulation mechanism, we exposed *w^1118^* and *Mgat1^1^/+* larvae to a second, distantly related, avirulent wasp from the family Braconidae, *Aphaereta sp.* [8]. *Aphaereta* eggs were encapsulated by *w^1118^* larvae (100% encapsulated, n = 79 eggs; Figure 3A). *Mgat1^1^/+* larvae were significantly impaired in encapsulation of *Aphaereta* eggs (16.2% encapsulated, n = 74 eggs, p<0.001 relative to control; Figure 3A). This demonstrates that the requirement for *Mgat1* is not specific to parasitoids from the family Figitidae, and suggests that it may be part of a more general wasp encapsulation response.

### The impaired encapsulation ability of *Mgat1* mutants correlates with decreased wasp resistance

To determine whether the impaired encapsulation phenotype seen in *Mgat1^1^/+* larvae represented failure of the immune response and reduced fly survival, we exposed *w^1118^* and *Mgat1^1^/+* larvae to the previously described avirulent wasps *L. clavipes* and *Aphaereta sp.*, allowed the larvae to develop to adulthood, and measured the eclosion rate of adult flies. We found that while *w^1118^* larvae developed into adult flies 100% of the time (n = 84 larvae; Figure 3D), only 34.3% of *Mgat1^1^/+* larvae developed into adult flies (n = 67 larvae, p<0.001 relative to control; Figure 3D), and the remainder eclosed as adult wasps. These data show an important role for *Mgat1* in wasp resistance.

### *Mgat1* is required in hemocytes for immune function

**Figure 4:**
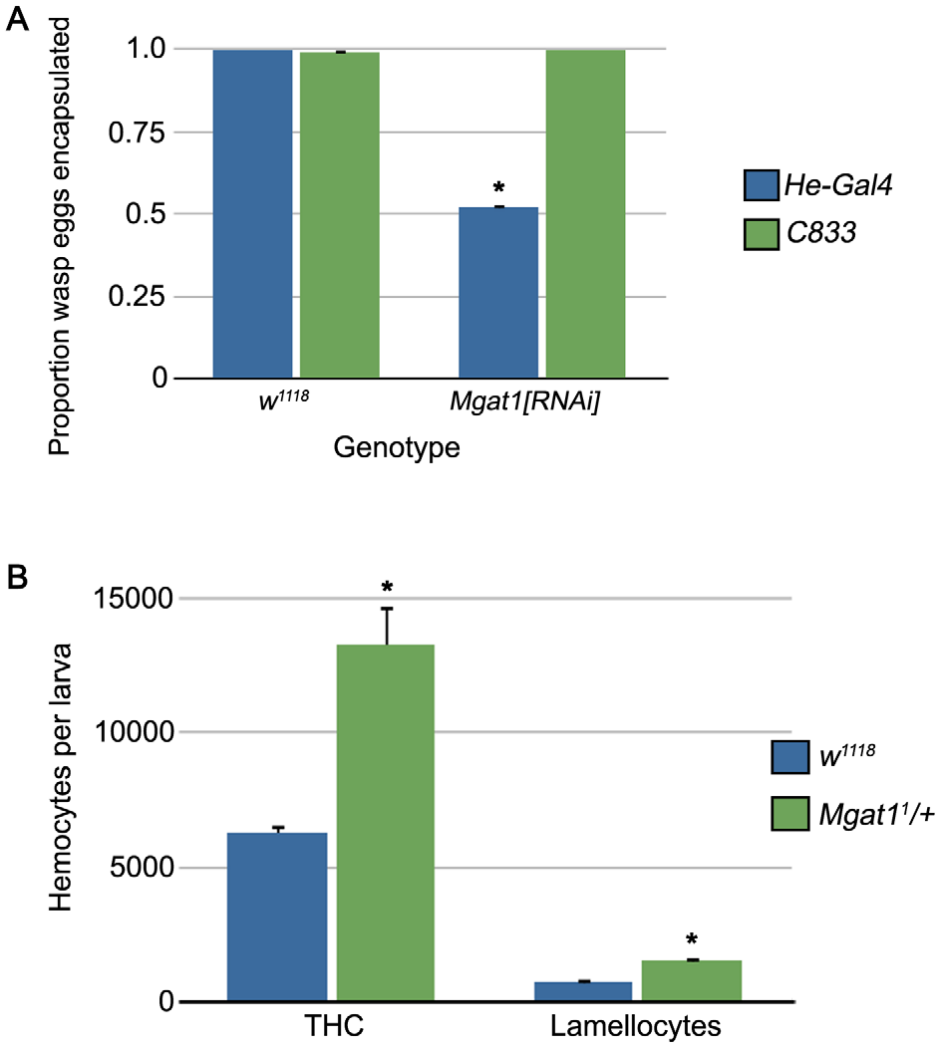
*Mgat1* is required in hemocytes, but loss of *Mgat1* does not result in reduced hemocyte numbers. (A) Proportion of wasp eggs encapsulated in the indicated genotypes crossed to hemocyte- (*He-Gal4*, blue bars) and fat body- (*C833*, green bars) specific Gal4 drivers. RNAi-mediated knockdown of *Mgat1* specifically in hemocytes led to a reduced encapsulation rate. (B) Circulating hemocyte counts in *L. clavipes* attacked *w^1118^* (blue bars) and *Mgat1^1^/+* (green bars) larvae. *Mgat1^1^/+* larvae showed comparable counts with control for both total hemocyte count (THC) and lamellocyte differentiation, suggesting that hematopoiesis occurs normally in these mutants. Error bars indicate standard error of the mean; * indicates p<0.01 relative to control.

The cellular encapsulation response is mediated by signaling in both of the main immune tissues in Drosophila, the hemocytes and fat body [15], [18], [45]. We used tissue specific RNA interference-mediated knockdown to determine where *Mgat1* is required. Using a previously characterized transgenic knockdown strain [40], we expressed dsRNA directed against *Mgat1* in hemocytes using the *He-Gal4* driver [46] and in the fat body using the *C833* driver [47]. When outcrossed to the *w^1118^* background control strain, both drivers were able to efficiently encapsulate *L. clavipes* eggs (*He-Gal4*: 100% encapsulated, n = 79 eggs; *C833*: 98.8% encapsulated, n = 86 eggs; Figure 4A). Knockdown of *Mgat1* in the hemocytes led to a significant reduction in encapsulation ability (51.8% encapsulated, n = 112 eggs, p<0.001 relative to control; Figure 4A). However, when *Mgat1* was knocked down in the fat body, encapsulation ability was unaffected (100% encapsulated, n = 81 eggs, p = 0.37 relative to control; Figure 4A). These findings demonstrate a hemocyte-specific role for *Mgat1* in cellular encapsulation.

### Hematopoiesis is not reduced in *Mgat1* mutants

The cellular encapsulation response is characterized by an increase in total hemocyte number and by the differentiation of lamellocytes [6]. Several studies have found that hemocyte number is an important determinant of wasp resistance, both within *D. melanogaster* and between Drosophila species [48], [49], [50]. To test whether loss of *Mgat1* led to defects in hematopoiesis, we collected hemolymph samples and measured both the total hemocyte count (THC) and number of lamellocytes in mutant and control larvae after 72 hour exposure to *L. clavipes*. We found that hematopoiesis was not reduced in *Mgat1^1^/+* larvae (Figure 4B). In fact, the THC of wasp attacked mutants was significantly higher than that seen in wasp attacked control larvae (THC: *w^1118^*, 6280 hemocytes per larva, *Mgat1^1^/+*, 13280 hemocytes per larva, p = 0.010). Furthermore, *Mgat1^1^/+* larvae had more circulating lamellocytes following wasp attack (*w^1118^*, 720 lamellocytes per larva, *Mgat1^1^/+*, 1520 lamellocytes per larva, p<0.001). These findings suggest that the failure to encapsulate wasp eggs observed in *Mgat1^1^/+* mutant larvae is not due to reduced hemocyte numbers, but instead must reflect a requirement for protein N-glycosylation in hemocyte function during capsule formation.

### *Mgat1* mutant larvae form defective capsules

**Figure 5:**
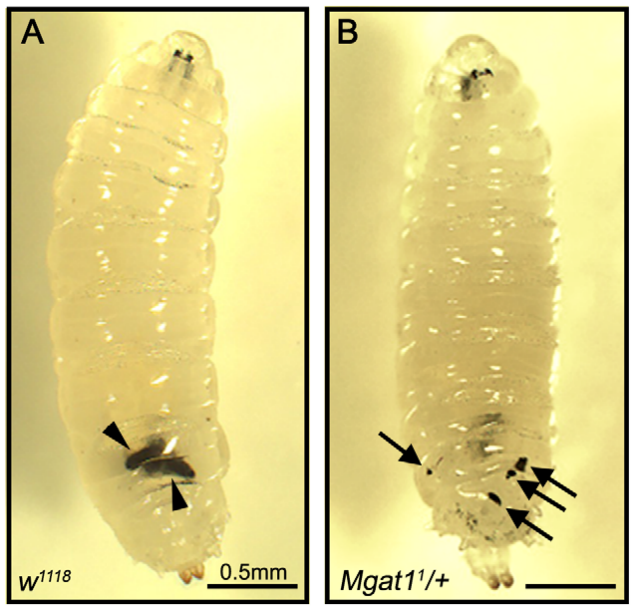
*Mgat1* mutants have a ‘broken capsule’ phenotype. *L. clavipes* attacked larvae imaged 48–72 hours PA. (A) *w^1118^* larvae successfully encapsulated *L. clavipes* eggs, indicated by arrowheads. (B) *Mgat1^1^/+* larvae had small pieces of melanized tissue floating within the hemocoel, indicated by arrows. Scale bars indicate 0.5 mm.

While scoring the egg encapsulation rate at 72 hours PA, we observed that whereas *w^1118^* attacked larvae contained encapsulated and melanized eggs (Figure 5A, indicated by arrowheads), 56.6% of attacked *Mgat1^1^/+* mutant larvae (n = 99 larvae) had pieces of melanized tissue floating in the hemocoel (Figure 5B, indicated by arrows). These melanized pieces appeared to be broken capsules and were specific to attacked larvae in which a hatched parasitoid larva was found. This broken capsule phenotype was also seen in 65.5% of larvae in which *Mgat1* was knocked down by dsRNA expression specifically in hemocytes (n = 87 larvae, data not shown), suggesting that loss of *Mgat1* in hemocytes is responsible for the appearance of broken capsules. The phenotype was seen at similar levels in six of the seven N-glycosylation pathway mutants that had a significant effect on encapsulation rate (*Ostγ*, *α-Man-I*, *α-Man-Ib*, *α-Man-II*, *α-Man-IIb*, and *FucT6*, data not shown; *OstΔ* mutants do not have the broken capsule phenotype suggesting that either this mutation effects encapsulation in a different way, or has pleiotropic effects that obscures the broken capsule phenotype). These data suggest that most N-glycosylation mutants can initiate capsule formation, but are in some way deficient in successfully encapsulating wasp eggs.

**Figure 6:**
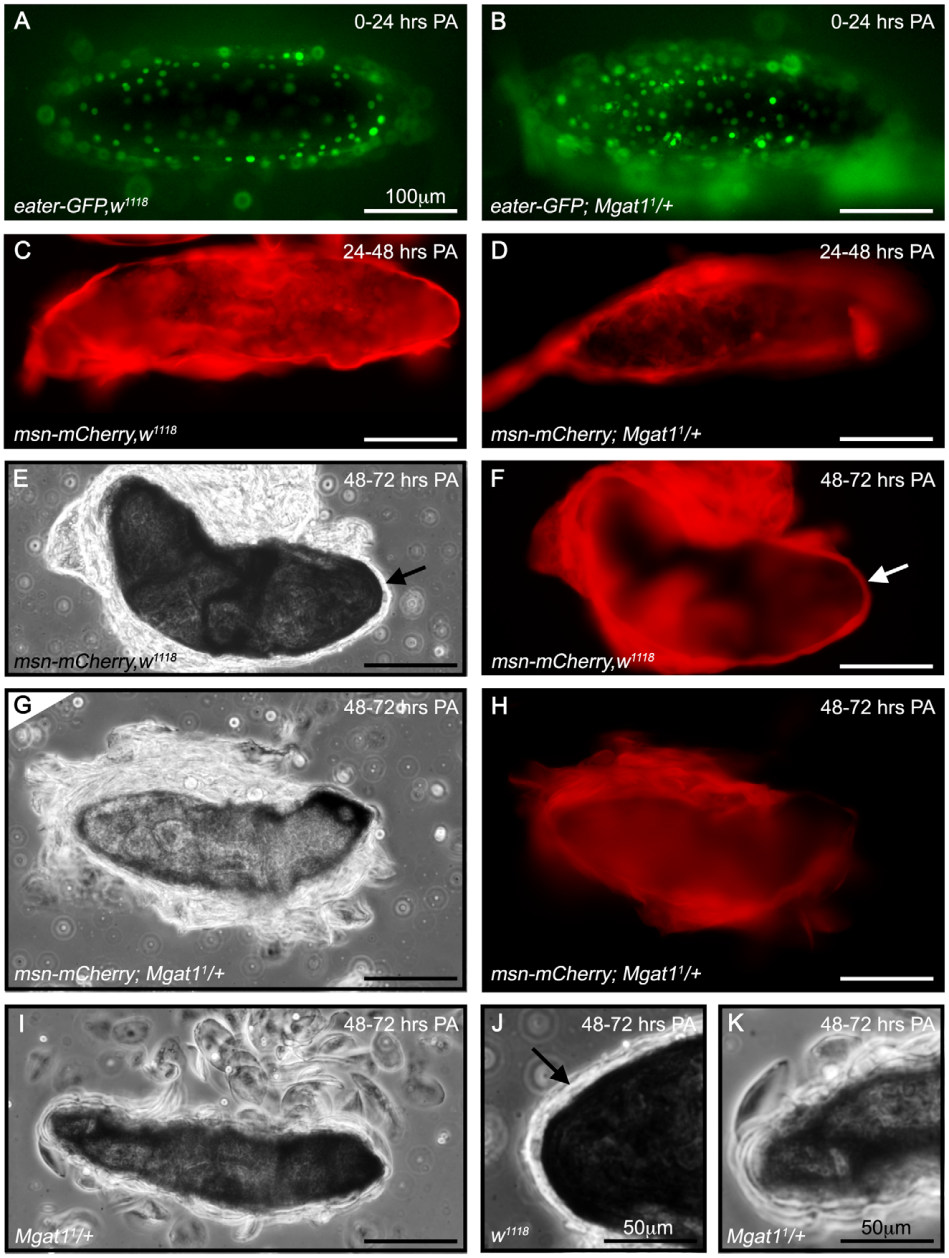
*Mgat1* mutants successfully initiate capsule formation but have a failure in lamellocyte consolidation. Brightfield and fluorescent images of *L. clavipes* eggs dissected from attacked fly larvae. (A,B) Plasmatocytes are marked by the expression of *eater-GFP* in *w^1118^* (A) and *Mgat1^1^/+* (B) backgrounds. At 0–24 hours PA, plasmatocytes in both genetic backgrounds migrated to, and surrounded, the wasp egg. (C–H) Lamellocytes are marked by the expression of *msn-mCherry*. At 24–48 hours PA, lamellocytes from both *w^1118^* (C) and *Mgat1^1^/+* (D) surrounded the wasp egg. By 48–72 hours PA, *w^1118^* larvae formed a consolidated lamellocyte layer around the wasp egg, indicated by arrows (brightfield in E, *mCherry* fluorescence in F). At the same time point, this consolidated layer was not seen in *Mgat1^1^/+* larvae, and instead individual lamellocytes were still visible (brightfield in G, *mCherry* fluorescence in H). (I) Additional brightfield image of a wasp egg from a *Mgat1^1^/+* larva that lacked a consolidated lamellocyte layer but was surrounded by individual lamellocytes. (J,K) Close up images of panels E and I (respectively) to focus on outer layer morphology of eggs at the 48–72 hour PA time point. A consolidated layer was consistently observed in *w^1118^* larvae (indicated by arrow in J) whereas in *Mgat1^1^/+* larvae (K), individual lamellocytes were clearly seen. Scale bars indicate 100 µm in A–I and 50 µm in J,K.

The major stages of capsule formation were observed using the hemocyte-subtype specific fluorescent markers *eater-GFP* to mark plasmatocytes and *msn-mCherry* to mark lamellocytes [51]. Following egg recognition, plasmatocytes adhere to the wasp egg forming the inner capsule layer [13]. In control larvae this process was completed during the first 24 hours PA (Figure 6A) and was not disrupted in *Mgat1^1^/+* larvae (Figure 6B). This inner plasmatocyte layer was then covered by several layers of lamellocytes [13] that formed during the 24–48 hour PA time point (Figure 6C). This process was also unaffected in *Mgat1^1^/+* larvae (Figure 6D), suggesting that egg recognition and cell migration are independent of protein N-glycosylation. Finally, the lamellocytes adhere to each other, forming a consolidated outer capsule [1], [13]. By 48–72 hours PA, this consolidated outer cell layer covering over the now melanized inner layer was observed in 100% of control larvae (n = 20, arrows, Figure 6E,F), and individual cells were no longer apparent. However, in *Mgat1^1^/+* larvae the lamellocyte layers did not consolidate and instead were observed as thick, loosely attached layers in which individual lamellocytes were still visible (Figure 6G–I). Closer examination revealed distinct outer layer morphologies, and a lack of consolidation in *Mgat1^1^/+* larvae (Figure 6J,K). We hypothesized that wasp larvae were capable of hatching from these loosely formed capsules, which then disintegrated into the small melanized pieces observed.

**Figure 7:**
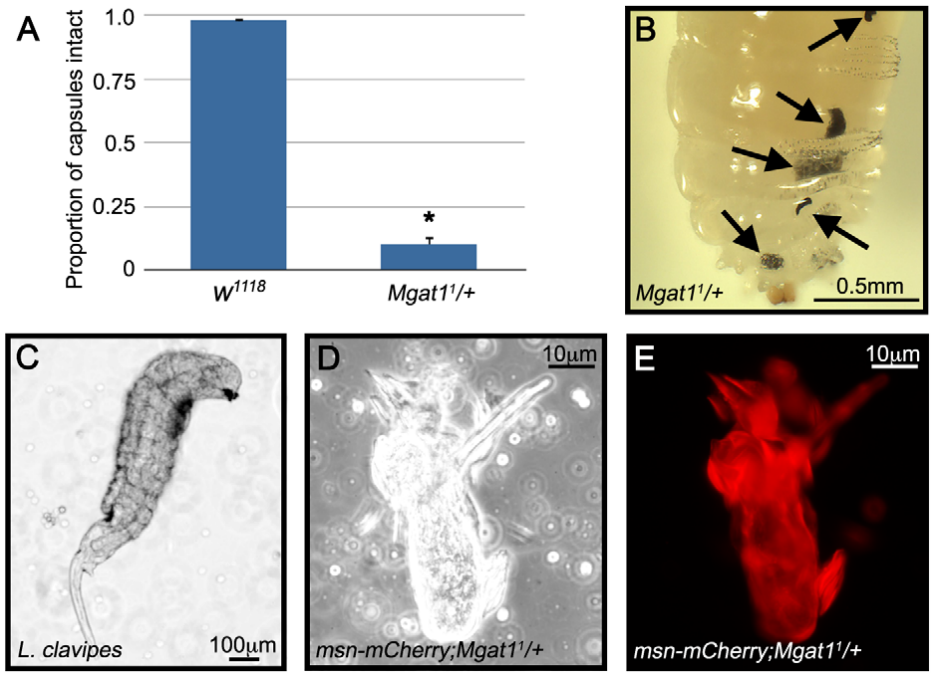
*Mgat1* mutant encapsulations break down over time. *L. clavipes* attacked larvae were scored for the presence of a melanized capsule at 24–48 hours PA, allowed to age a further 24 hours, then scored again. (A) Proportion of *w^1118^* (blue bars) and *Mgat1^1^/+* (green bars) capsules that remained intact at 48–72 hours PA. Error bars indicate standard error of the mean; * indicates p<0.01 relative to control. (B) Brightfield image of a *Mgat1^1^/+* larva with broken capsule pieces indicated by arrows. (C) All of the *Mgat1^1^/+* larvae with this phenotype contained live wasp larvae. (D,E) Brightfield and fluorescent images of a broken capsule piece from a *msn-mCherry;Mgat1^1^/+* larva. The piece was composed largely of *mCherry* positive lamellocytes. Scale bars indicate 0.5 mm in B, 100 µm in C and 10 µm in D,E.

Because defective capsules in *Mgat1^1^/+* larvae were melanized, and therefore visible in live larvae, we were able to follow the progression of capsule formation and disintegration in control and *Mgat1^1^/+* larvae. At 24–48 hours PA, we found that there was no significant difference in the incidence of capsule formation in the two genotypes; 94.8% of attacked *w^1118^* larvae (n = 231) and 79.2% of attacked *Mgat1^1^/+* larvae (n = 101, p = 0.09) showed evidence of capsule formation. To trace the fate of the capsules, these larvae were dissected at 48–72 hours PA. We found that whereas 98.2% of the *w^1118^* capsules remained whole (n = 219; Figure 7A), only 10.0% of *Mgat1^1^/+* capsules were recovered intact (n = 80, p<0.001 relative to control; Figure 7A), and the remainder of the mutant larvae showed the broken capsule phenotype (Figure 7B) and contained a live wasp larva (Figure 7C). Examination of the melanized pieces using the *msn-mCherry* marker demonstrated that they were composed of lamellocytes as would be expected of a broken capsule (Figure 7D,E). This analysis demonstrates that protein N-glycosylation is required for completion of capsule formation and suggests that melanization alone is not sufficient for parasite-killing, but must be accompanied by lamellocyte consolidation.

### Lamellocyte protein N-glycosylation is inhibited by the parasitoid wasp *L. victoriae*

**Figure 8:**
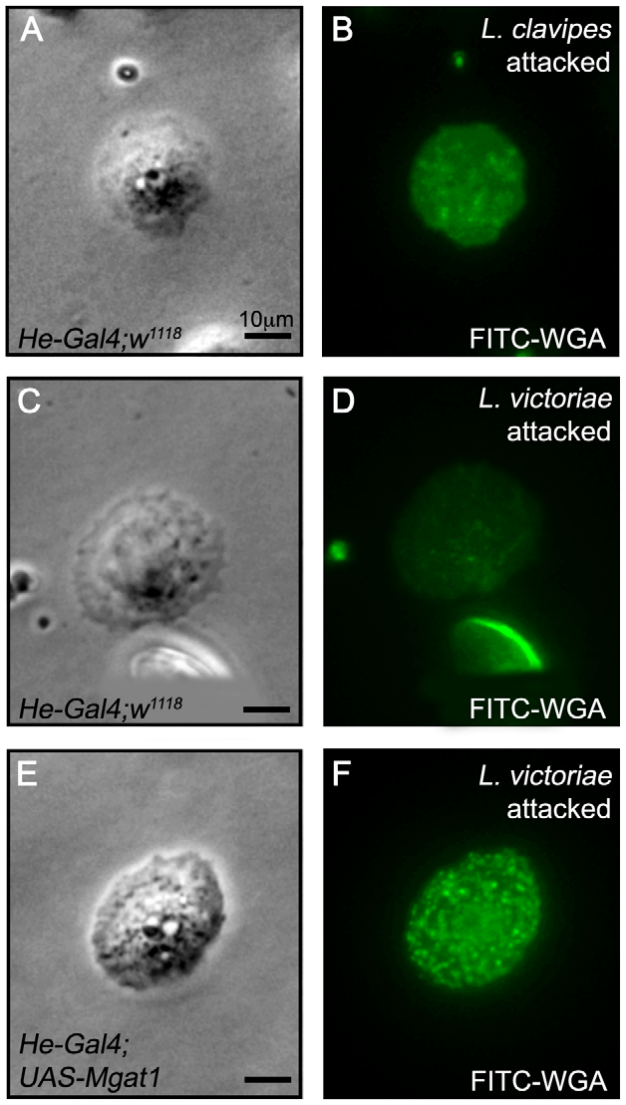
Lamellocyte WGA staining was reduced in *L. victoriae* attacked larvae and restored by *Mgat1* overexpression. Paired brightfield and FITC images of hemocytes stained with FITC-WGA 24–48 hours following attack by the indicated wasp. (A–D) Lamellocytes from *He-Gal4;w^1118^* larvae were WGA+ following *L. clavipes* attack (A,B), but were WGA− following *L. victoriae* attack (C,D). (E,F) The WGA+ staining was restored to *L. victoriae* attacked larvae by hemocyte specific expression of *Mgat1* in *He-Gal4;UAS-Mgat1* larvae. Scale bars indicate 10 µm.

Since *Mgat1*-dependent protein N-glycosylation was required for wasp resistance, we hypothesized that wasp venom may target the protein N-glycosylation pathway as a virulence mechanism. We previously observed that larvae attacked by the parasitoid wasp *L. victoriae* occasionally show a broken capsule phenotype similar to that seen in *Mgat1* mutants (see below). We therefore assayed lamellocyte glycosylation in *L. victoriae* attacked larvae to test whether *L. victoriae* attack altered the WGA staining pattern. We found that these larvae showed decreased lamellocyte WGA staining (compare Figure 8A,B to 8C,D), similar to that seen in *Mgat1* mutants. This suggests that *L. victoriae* venom either directly targets the N-glycosylation pathway or indirectly disrupts protein glycosylation, perhaps by altering host carbohydrate metabolism or upstream signaling events. If the venom acts directly on glycosylation, then ectopic expression of *Mgat1* might be predicted to rescue the WGA staining phenotype. Hemocyte-specific expression of full length *Mgat1* [40] was sufficient to restore the lamellocyte WGA+ pattern in *L. victoriae* attacked larvae (Figure 8E,F), suggesting *L. victoriae* venom directly affects N-glycosylation.

### Hemocyte-specific overexpression of *Mgat1* increased encapsulation of *L. victoriae* eggs

**Figure 9:**
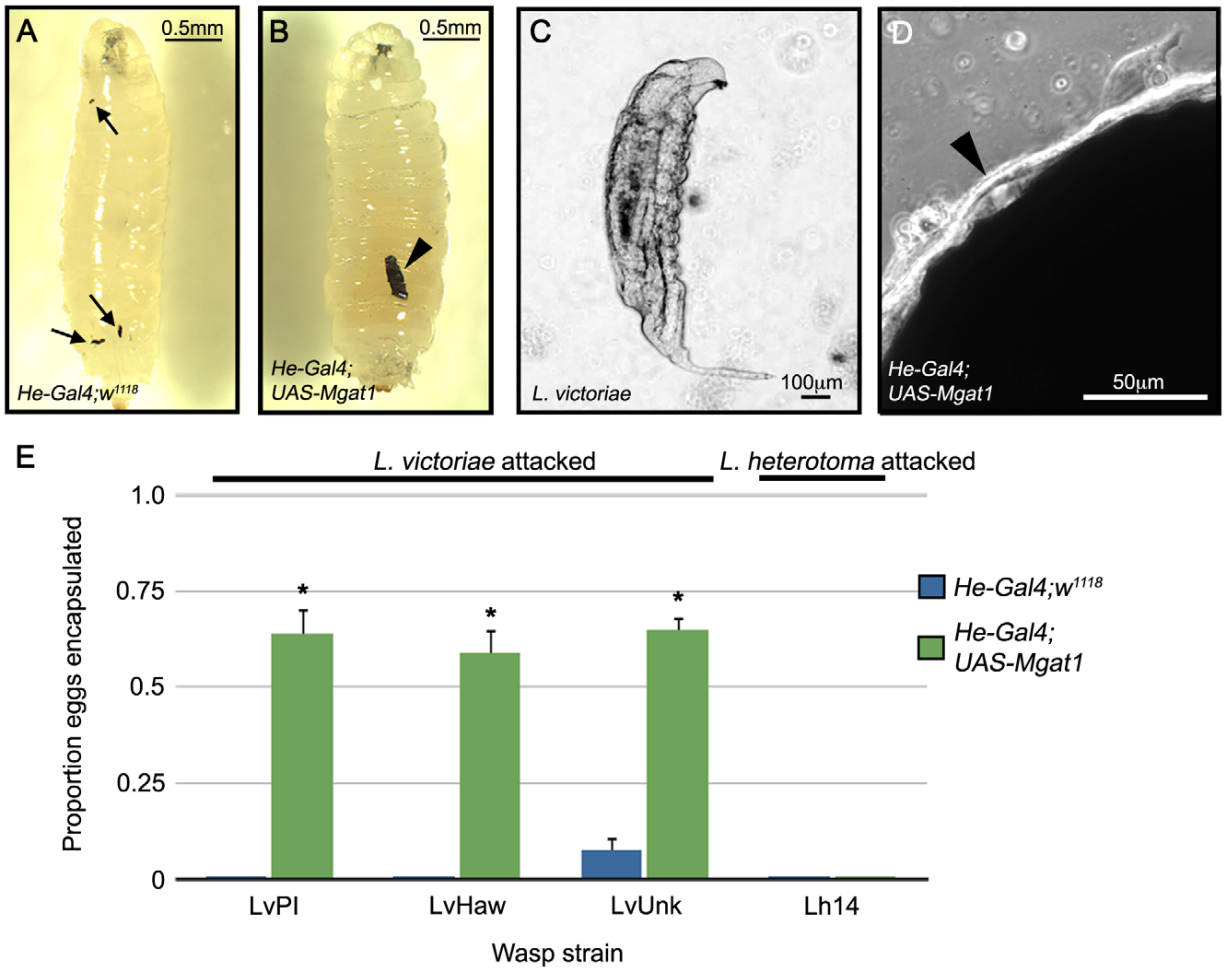
Hemocyte specific *Mgat1* overexpression conferred resistance to *L. victoriae*. (A–D) Brightfield images of *L. victoriae* attacked larvae 48–72 hours PA. (A,C) Control *He-Gal4;w^1118^* larvae showed the broken capsule phenotype, indicated by arrows (A). Live *L. victoriae* larvae were recovered from *He-Gal4;w^1118^* larvae with the broken capsule phenotype (C). (B,D) In *He-Gal4;UAS-Mgat1* larvae encapsulated *L. victoriae* eggs were observed, indicated by the arrowhead (B). A consolidated lamellocyte layer (arrowhead in D) was observed in *Mgat1* over-expressing larvae. (E) Proportion of eggs encapsulated following attack by the indicated wasp strains. *He-Gal4;w^1118^* larvae (blue bars) did not encapsulate the eggs of any of the *L. victoriae* strains (left) or *L. heterotoma* (right). Overexpression of *Mgat1* in *He-Gal4;UAS-Mgat1* larvae (green bars) conferred the ability to encapsulate *L. victoriae* (left) but not *L. heterotoma* eggs (right). Error bars indicate standard error of the mean; * indicates p<0.01 relative to paired control. Scale bars indicate 0.5 mm in A,B, 100 µm in C and 50 µm in D.

We found that 19.5% of *L. victoriae* attacked larvae (n = 118) showed the broken capsule phenotype (Figure 9A) and that the appearance of broken capsules was correlated with the presence of live wasp larvae in the larval hemocoel (Figure 9C). This suggests that, like in *Mgat1* mutants, the broken capsule seen in *L. victoriae* attacked larvae may be indicative of a failed encapsulation response.

Since expression of *Mgat1* in hemocytes restored WGA staining in *L. victoriae* attacked larvae, we tested to see whether it would also block the immune suppressive effects of *L. victoriae* venom. We first verified that *Mgat1* overexpression did not impair cellular encapsulation ability (97.4% of *L. clavipes* eggs encapsulated, n = 39 eggs). We then assayed the ability of control and *Mgat1* over-expressing larvae to encapsulate the eggs of three strains of *L. victoriae* (LvPhil, LvHaw and LvUnk). All three were virulent on control larvae: in both LvPhil and LvHaw attacked larvae, 0% of the eggs were encapsulated (n = 84 and 91 respectively), and in LvUnk attacked larvae, 7.4% of the eggs were encapsulated (n = 95; Figure 9E).

The broken capsule phenotype was suppressed by hemocyte-specific *Mgat1* overexpression (0% of attacked larvae had broken capsules, n = 120, p<0.001 relative to control), which conferred upon larvae the ability to encapsulate *L. victoriae* eggs (Figure 9B). Accordingly, we observed that these larvae formed a consolidated lamellocyte layer (arrowhead, Figure 9D). Furthermore, this *L. victoriae* egg encapsulation happened at high frequency in all three tested strains: in LvPhil attacked larvae, 64.3% of the eggs were encapsulated (n = 98, p<0.001 relative to control), in LvHaw attacked larvae, 59.2% of the eggs were encapsulated (n = 98, p<0.001 relative to control), and in LvUnk attacked larvae, 64.7% of the eggs were encapsulated (n = 99, p<0.001 relative to control; Figure 9E). This suggests that targeting protein N-glycosylation is a conserved virulence mechanism in *L. victoriae* from diverse populations.

The ability of fly larvae with hemocyte specific *Mgat1* overexpression to encapsulate *L. victoriae* eggs suggests that decreasing glycosylation (as reflected in decreased WGA staining) may be an important aspect of *L. victoriae* virulence. Based on our results we hypothesize that *L. victoriae* venom may contain ‘anti-glycosylation’ factors that either block protein N-glycosylation (directly or indirectly as described above), or de-glycosylate membrane surface proteins. We would further predict that since this venom activity is sensitive to GnTI activity, it likely targets the N-glycosylation pathway at, or downstream of, the level of *Mgat1* function.

To test the specificity of this mechanism, we next assayed encapsulation rates using the sister species to *L. victoriae*, *L. heterotoma* [8]. Neither control nor *Mgat1* over-expressing larvae were able to encapsulate *L. heterotoma* eggs (control: 0% encapsulation, n = 90 eggs; *Mgat1* overexpression: 0% encapsulation, n = 88 eggs; Figure 9E). *L. heterotoma* has previously been demonstrated to induce lysis of Drosophila lamellocytes [52], presumably removing any need for an ‘anti-glycosylation’ role of its venom. This finding demonstrates that the enhanced encapsulation ability conferred by ectopic *Mgat1* expression is not generalized to all wasp parasites and that wasp virulence strategies differ greatly even among closely related species.

### Conclusions

The findings described in this report clearly demonstrate an important role for protein N-glycosylation in the Drosophila cellular encapsulation response against parasitoid wasp eggs, and specifically in the consolidation of the outer lamellocyte layers of melanotic capsules. The identities and functions of the relevant glycosylated protein(s) are still unknown, so further study will be required to uncover the exact molecular mechanisms responsible for lamellocyte consolidation. Furthermore, we have uncovered a parasite virulence strategy that is dependent on prevention of lamellocyte protein N-glycosylation and lamellocyte consolidation.

Protein N-glycosylation is also a common post-translational modification in human immune cells [53] and in fact many key players in both innate and adaptive immune responses are glycoproteins [54]. It has been demonstrated that protein N-glycosylation is important for regulating protein stability, and for the activation of numerous immune receptors, including the B- and T-cell receptors, along with cytokine and Toll-like receptors [54], [55]. During an immune response, N-glycosylated proteins play important roles in pathogen recognition and in mediating cell-cell interactions among cells of the immune system [55], [56], perhaps in a manner related to the consolidation of Drosophila lamellocytes.

Recent findings have revealed that the Drosophila cellular encapsulation process may serve as a useful model of human immune cell function. It is becoming increasingly apparent that the molecular mechanisms underlying cellular encapsulation in Drosophila are highly homologous to those involved in important human immune functions such as immune cell adhesion, wound healing, thrombosis, and inflammation [16], [57], [58]. Based on this mechanistic homology and on a shared requirement for protein N-glycosylation [59], the cellular encapsulation of parasitoid wasp eggs may serve as a novel model to further examine the conserved roles of N-linked protein glycosylation in immunity.

## Materials and Methods

### Insect strains

The following *Drosophila melanogaster* alleles were used in this study: *y,w;CG6370^DG02210^* (insertion of a *P{wHy}* element into exon 1 of the locus [60]), *y,w;CG7830^EY16757^* (insertion of *P{EPgy2}* into exon of the locus 1 [61]), *y,CG1518^KG03333^* (insertion of *P{SUPor-P}* into exon 1 of the locus [61]), *y,α-Man-I^KG04725^* (insertion of *P{SUPor-P}* into intron 1 of the locus [61]), *w^1118^*; *CG11874^f07221^* (insertion of *PBac{WH}* into exon 2 of the locus [61], [62]), *w^1118^*; *α-Man-II^G4901^* (insertion of *P{EP}* into exon 1 of the locus [61], [63]), *y,w;α-Man-IIb^KG05078^* (insertion of *P{SUPor-P}* into intron 1 of the locus [61]), *y,w;Mgat2^EY07798^* (insertion of *P{EPgy2}* into exon 1 of the locus [61]), *w^1118^*,*FucT6^e02394^* (insertion of *PBac{RB}* into exon 4 of the locus [61], [62], [64]); all from the Bloomington Drosophila Stock Center), and *w^1118^*;*Mgat1^1^* (provided by G. Boulianne [31]). The *w^1118^*, *y,w* and *y;ry* strains served as genetic background controls throughout the study. Overexpression and RNAi knockdown experiments were performed with the Gal-4 drivers *He-Gal4* [46] and *C833* [47] (both from the Bloomington Drosophila Stock Center), and the previously described *Mgat1* reagents *UAS-Mgat1* and *UAS-Mgat1^RNAi^* (provided by G. Boulianne [40]). *msn-mCherry* and *eater-GFP* (provided by R. Schulz [51]) were used to mark lamellocytes and plasmatocytes, respectively.

In this study we used the figitid wasp species *L. clavipes*, *L. victoriae*, and *L. heterotoma*, and the braconid wasp *Aphaereta sp.* The *L. clavipes* strain used in this study was provided by J. van Alphen, and the *L. victoriae* strain LvUnk was provided by S. Govind. The other *L. victoriae* strains were collected by the Schlenke lab (LvHaw in Hawaii and LvPhil in the Philippines), as was the strain of *Aphaereta sp.* (collected in Atlanta, GA). The *L. heterotoma* strain used has been previously described [18]. The laboratory culture of *L. heterotoma* is maintained on *D. melanogaster*, *L. victoriae* is maintained on *D. annanassae* and *Aphaereta sp.* and *L. clavipes* are both maintained on *D. virilis*.

### Wasp exposure

Adult females of each fly strain were allowed to lay eggs onto molasses medium supplemented with yeast paste in 60 mm Petri dishes. After 96 hours, adult flies were removed and second instar fly larvae were collected for wasp exposure. Forty fly larvae were moved into a 35 mm Petri dish filled with 1 mL of Drosophila medium. Three female wasps were then placed onto the dish and allowed to attack for either 24 or 72 hours depending on the experiment (described below). The 72 hour wasp exposure condition allowed for nearly all larvae to be attacked without significant rates of superparasitism. Attack rates for each wasp species were calculated based on three replicates of *w^1118^* wasp exposures, and were as follows: *L. clavipes*: 1.00±0.03 eggs/larva, *Aphaereta sp.*: 0.88±0.05 eggs/larva, *L. victoriae* (average of all three strains): 0.99±0.06 eggs/larva and *Lh14*: 1.02±0.08 eggs/larva. Unattacked controls were treated in parallel.

### WGA staining

For WGA staining, larvae were exposed for 24 hours and dissected at the indicated times. Larvae were bled onto diagnostic slides (Tekdon, Inc.) and hemocytes were allowed to adhere for 5 minutes. Hemocytes were then stained with 100 µg/ml FITC labeled WGA from Vector Laboratories (FL-1021) for 3 minutes and washed three times with Drosophila Ringer's solution as described in [22]. Stained hemocytes were visualized using an Olympus BX51 microscope with a FITC filter and Olympus DP2-BSW software.

### Encapsulation rate

After a 72 hour wasp exposure, thirty fly larvae from each attack plate were dissected to assay attack rate and encapsulation rate. At the end of the exposure period larvae were scored for the presence of an encapsulated wasp egg or live wasp larva. Experiments were performed in triplicate.

### Eclosion assay

After a 72 hour wasp exposure, thirty fly larvae were recovered from each plate and allowed to eclose. The total number of flies and wasps eclosed from the treatments were determined 7 and 14 days following infection, respectively. By these times, all viable flies and wasps emerged. Experiments were performed in triplicate.

### Hemocyte counts

After a 72 hour wasp exposure, five larvae from each plate were removed, rinsed in Drosophila Ringer's solution and bled into 20 µl of 1 µl PBS containing 0.01% phenylthiourea (PTU). Hemocytes were then pipetted into a disposable hemocytometer (Incyto C-Chip DHC-N01) and allowed to adhere for 30 minutes. Hemocytes from each sample were counted from sixteen 0.25×0.25×0.1 mm squares. Counts were then normalized to a per larva value. Experiments were performed in triplicate.

### Imaging

For whole larva imaging, larvae were chilled and imaged using a Leica stereo-dissecting scope with a Moticam MIP 2.0 and Multi-Focus Pro software. For imaging capsules, larvae were dissected into Drosophila Ringer's solution or PBS containing 0.01% PTU (PTU was found to quench fluorescence and so was only used for brightfield imaging). Dissected capsules were visualized using an Olympus BX51 microscope with FITC and TRITC filters with Olympus DP2-BSW software. Figures were compiled using Adobe Photoshop.

### Capsule fate assay

Larvae were exposed to wasps for 24 hours, and then aged a further 24 hours. At this time point larvae were scored for the presence of a capsule in the hemocoel. Larvae with capsules were selected and aged for a further 24 hours. At this time point the larvae were scored for the presence of an encapsulated wasp egg or broken capsule and live wasp larva. Experiments were performed in triplicate.

### Data analysis

For analysis of encapsulation/eclosion rates and phenotypic penetrance, mutant strains were compared to wild type background controls by Student's t-test (Numbers) using data from independent replicates.
